# What factors shape quality of life for women affected by gynaecological cancer in South, South East and East Asian countries? A critical review

**DOI:** 10.1186/s12978-022-01369-y

**Published:** 2022-03-19

**Authors:** Belinda Rina Marie Spagnoletti, Linda Rae Bennett, Christina Keenan, Suman Surendra Shetty, Lenore Manderson, Barbara McPake, Siswanto Agus Wilopo

**Affiliations:** 1 Nossal Institute for Global Health, Melbourne School of Population and Global Health, University of Melbourne, Melbourne, Australia; 2 Center for Reproductive Health, Faculty of Medicine, Public Health and Nursing, Universitas Gadjah Mada, Yogyakarta, Indonesia; 3 School of Public Health, University of the Witwatersrand, Johannesburg, South Africa; 4 School of Social Sciences, Monash University, Clayton, Australia

**Keywords:** Health-related quality of life, Patient experiences, Female cancer, Sexuality, Mental health, Psychosocial support, Physical health

## Abstract

**Background:**

Gynaecological cancers are among the most prevalent cancers worldwide, with profound effects on the lives of women and their families. In this critical review, we explore the impacts of these cancers on quality of life (QOL) of women in Asian countries, and highlight areas for future inquiry.

**Methods:**

A systematic search of the literature was conducted in six electronic databases: Web of Science, Scopus, Global Health (CAB Direct), PsycINFO (Ovid), EBMR (Ovid), and Medline (Ovid). Screening resulted in the inclusion of 53 relevant articles reporting on 48 studies.

**Results:**

Most studies were conducted in high and upper-middle income countries in East Asia and used quantitative approaches. Women had predominantly been diagnosed with cervical or ovarian cancer, and most had completed treatment. Four key interrelated domains emerged as most relevant in shaping QOL of women affected by gynaecological cancer: support, including identified needs, sources and forms; mental health, covering psychological distress associated with cancer, risk and protective factors, and coping strategies; sexual function and sexuality, focused on physiological, emotional and relational changes caused by gynaecological cancers and treatments, and the impacts of these on women’s identities; and physical health, covering the physical conditions associated with gynaecological cancers and their impacts on women’s daily activities.

**Conclusion:**

QOL of women affected by gynaecological cancer is shaped by their mental and physical health, support, and changes in sexual function and sexuality. The limited number of studies from lower- and middle-income countries in South and Southeast Asia highlights important knowledge gaps requiring future research.

## Plain language summary

Multiple factors shape the quality of life of women affected by gynaecological cancers in Asian countries as elsewhere. We identified 53 articles reporting on 48 studies, most conducted in high- and upper-middle income East Asian countries, with much less attention to women in lower income countries in South and Southeast Asia. Most studies used quantitative research methods to gain an understanding of the impact on women diagnosed with cervical or ovarian cancer who had completed treatment. Women’s quality of life was shaped by their mental and physical health, their support needs, and the changes they experienced in sexual function and sexuality.

## Background

In 2020, the International Agency for Research on Cancer reported that gynaecological cancers represented 7.29% of all new cancer cases globally, with 1,398,600 women newly diagnosed in the preceding year [1]. These cancers accounted for 6.74% of all female cancer mortalities in the same period [1]. Given poor survival rates, researchers have focused on survival and recovery. However, Quality of Life (hereafter, QOL) is important throughout treatment, regardless of prognosis. Different treatments for gynaecological cancers impact QOL, shaping treatment decisions and influencing the support needs of women [2]. Most research has been conducted in the Global North, with women who embrace Western cultural identities and values. Yet over 60% of the global population resides in the Asian region, and approximately two-thirds of Asian nations are classified as low- and middle-income countries [3]. In the following, we explore what is known about QOL among women in Asian countries, identify gaps in knowledge, and propose research priorities to inform appropriate interventions to enhance QOL.

Cervical cancer is responsible for over 50% of all gynaecological cancers globally; this is followed by ovarian and uterine cancers, most often endometrial [4]. Vaginal cancer and cancer of the vulva are less common. In Asian countries, breast cancer is predominant among women, but the second most common cancer is cervical cancer, then uterine and ovarian cancers [5]. All women are at risk of developing gynaecological cancers, with risk increasing with age [6]. Cervical cancer incidence rates are higher in South and Southeast Asian countries (19.3/100,000 and 16.3/100,000, respectively), compared with Eastern (7.9/100,000) and Western (4.4/100,000) Asian countries [5].

The concepts of QOL and health-related QOL are often used to discern the impact of gynaecological cancers on women [7, 8]. Health-related QOL has been defined as a multidimensional construct, ideally determined using different instruments to assess physical, emotional, social, and cognitive functions, as well as pain, discomfort, and other symptoms [9, 10]. Cervical cancer, including disease symptoms, treatment side-effects and toxicity, and short-term psychological effects, influence individual functioning and wellbeing: this includes physical, emotional, cognitive, and social aspects, sexual and body image, role, spirituality and financial status. These domains contribute to shape women’s ‘global’ or overall QOL, wellbeing and happiness. Global QOL is also influenced by contextual factors, including household finances, work, safety, family and culture.

The suitability of tools and their psychometric qualities for measuring QOL related to gynaecological cancers has been explored in several recent reviews [11–14], predominantly in China [15–20]. Ding and colleagues found reasonable internal consistency and predictive and divergent validity within the Chinese version of the Sense of Coherence Scale [16], but noted that the concept of coherence may have a different meaning among Chinese people, and recommended further psychometric evaluation and longitudinal studies to ascertain generalisability and stability [16]. Luckett and colleagues maintain that QOL is circumstantial, given that the instruments are only valid in the context of specific populations and treatments [11], and Zeng and colleagues have argued for future studies to explore cervical cancer survivors’ QOL using culturally-grounded instruments [14]. Reflecting on the Chinese context and the influences of Confucianism, Taoism and traditional Chinese medicine, researchers have reinforced the subjective cultural basis of what constitutes ‘normality’ and health, which influences perceptions of and responses to measuring QOL [14, 21]. The impact of gynaecological cancers on kinship, familial relationships and gender roles may also influence Chinese women’s QOL [14]. Further, sexuality is a key dimension of QOL, but this is rarely explored. This suggests the value of ethnographic or other qualitative approaches to be used with standardised instruments to determine the effects of gynaecological cancer on women’s sexuality.

In the studies included in this review, 44 standardised instruments were utilised across 36 articles (see Table 1), resulting in methodological and conceptual challenges in analysing QOL scores. We could not be confident in comparing QOL scores produced using the same scales but in different populations, with women affected by different types of gynaecological cancer, who had undergone different treatment regimens, or who were at different stages of their illness trajectories. Given this, rather than comparing results of QOL scales, we undertook a critical thematic analysis of findings across key domains of QOL in the selected studies.

**Table 1:** Standardised instruments and scales applied in 36 articles

Instrument / scale name	Number of articles applied in	Applied in articles
European Organization for Research and Treatment of Cancer Quality of Life Questionnaire 30 Version 3 (EORTC QLQ-C30)	11	[43, 45, 49, 60, 69–71, 75, 79, 81, 83]
Hospital Anxiety and Depression Scale (HADS)	9	[33, 49, 56, 57, 59, 60, 62–64]
European Organization for Research and Treatment of Cancer Quality of Life Cervical Cancer Module 24 (EORTC QLQ‐CX24)	5	[45, 60, 69, 75, 79]
Female Sexual Function Index (FSFI)	4	[32, 73, 74, 80]
Functional Assessment of Cancer Therapy-General Version 4 (FACT-G)	4	[33, 57, 61, 68]
Sexual Function—Vaginal Changes Questionnaire (SVQ)	2	[33, 58]
Medical Outcomes Study Short Form-36 (MOS SF-36)	2	[58, 65]
Mishel’s Uncertainty in Illness Scale (MUIS)	2	[33, 56]
Medical Outcomes Study Social Support Survey (MOS-SSS)	2	[33, 70]
World Health Organization Quality of Life Scale (WHOQOL-BREF)	2	[21, 40]
Psychosocial Adjustment to Illness Scale (PAIS) or PAIS Self-Report (PAIS-SR)	2	[21, 58]
Rosenberg Self-Esteem Scale (SES)	2	[57, 70]
Perceived Social Support Scale (PSSS)	2	[42, 49]
Functional Assessment of Cancer Therapy-Cervix Questionnaire (FACT-CX)	2	[52, 73]
Herth Hope Index (HHI)	2	[62, 64]
European Organization for Research and Treatment of Cancer Quality of Life Ovarian Cancer Module 28 (EORTC-QLQ-OV28)	2	[43, 49]
Sexual Activity Questionnaire (SAQ)	1	[79]
Profile of Mood States (POMS)	1	[21]
Positive & Negative Affect Schedule (PANAS)	1	[72]
Ruminative Responses Scale (RRS)	1	[72]
Emotion Regulation Questionnaire (ERQ)	1	[72]
Derogatis Sexual Functioning Inventory (DSFI)	1	[76]
Mastery Scale (MS)	1	[76]
Brief Resilience Coping Scale (BRCS)	1	[76]
Social Support Scale (SSS)	1	[76]
Body Image Scale (BIS)	1	[68]
Functional Assessment of Cancer Therapy—Ovary (FACT-O)	1	[42]
Functional Assessment of Chronic Illness Therapy-Spiritual Well-being Scale (FACT-Sp)	1	[73]
Hamilton Anxiety Scale (HAM-A)	1	[53]
Perceived Stress Scale (PSS-10)	1	[62]
Resilience Scale (RS)	1	[62]
EuroQoL (EQ-5D-3L)	1	[67]
Sexual Function After Gynaecologic Illness Scale (SFAGIS)	1	[58]
Symptom Distress Scale (SDS)	1	[71]
Sense of Coherence Scale 13 (SOC-13)	1	[52]
Life Orientation Scale-Revised (LOT-R)	1	[64]
General Self-efficacy Scale (GSES)	1	[64]
National Health and Social Life Survey (NHSLS)	1	[69]
McGill Quality of Life Questionnaire (MQOL)	1	[60]
Interpersonal Support Evaluation List (ISEL)	1	[66]
Fear of Cancer Recurrence Inventory (FCRI)	1	[66]
Memorial Symptom Assessment Scale-Short Form (MSAS-SF)	1	[61]
Type-D Personality Scale-14 (DS14)	1	[61]
M. D. Anderson Symptom Inventory (MDASI)	1	[82]

In this review, we determine the geographic reach of recent research in Asia and summarise what is known of QOL among women diagnosed with gynaecological cancers. We then describe differences in investigating QOL according to the form and stage of gynaecological cancer. We also identify contextual social and cultural factors that might influence QOL.

## Methods

### Approach

**Fig. 1:**
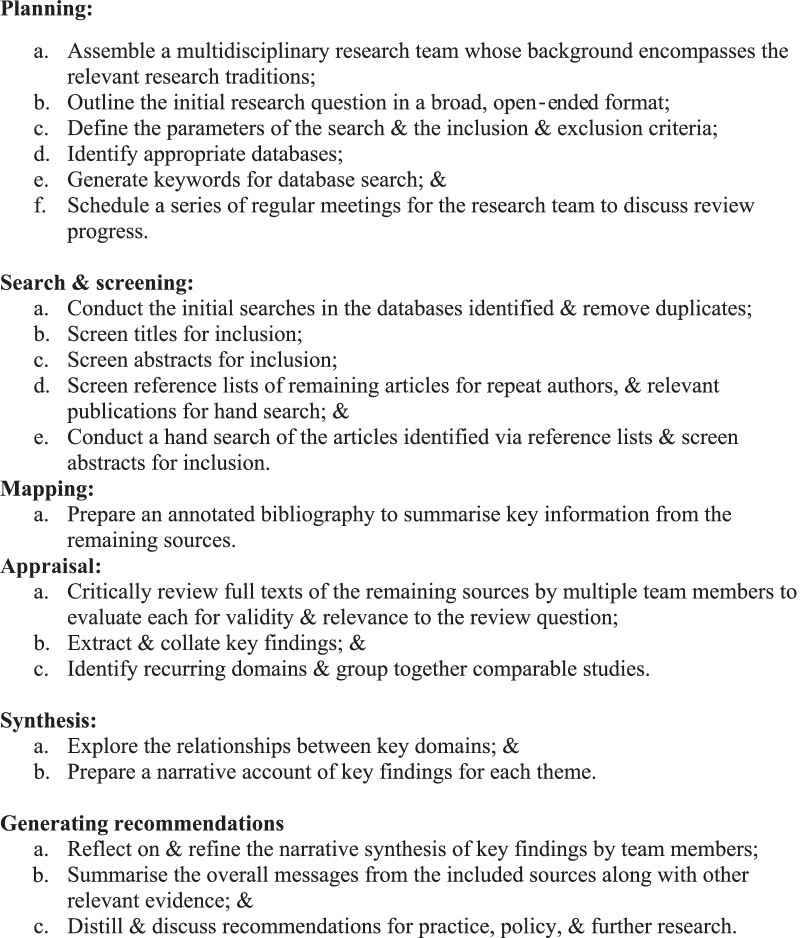
Phases in developing a narrative synthesis of QOL among women affected by gynecological cancers in Asia. Adapted from Greenhalgh and colleagues [24]

We systematically identified, screened and determined the eligibility of literature concerning QOL of women affected by gynaecological cancer, and employed narrative synthesis to explore and analyse key themes and findings from the articles that met our eligibility criteria [22]. Given the limitations associated with the standardised tools that measure gynaecological cancer-related QOL, we sought to extract and explore the key themes and findings from studies with heterogenous approaches, methods and foci. Narrative synthesis has been described as a storytelling approach utilised by researchers to generate new insights, by systematically and transparently integrating findings from research with diverse methodological and epistemological approaches [22–24]. It involves drawing from a heterogenous body of literature to identify key themes, distill overlapping and contrasting findings, and develop a narrative that encompasses these themes and findings [22]. We determined that the narrative synthesis approach was best aligned with the objectives of our review, allowing us to distinguish the factors influencing QOL, and distill and build a narrative of the commonalities and variances associated with women’s country or place of residence, social and cultural factors, and type and stage of cancer. Our process is outlined in Fig. 1.

### Data sources and search strategy

An online search for English-language peer-reviewed journal articles published from January 2000 to April 2020 on women’s experiences of gynaecological cancers and their QOL in Asian countries was conducted by CK. The search was confined geographically to countries in South, Southeast or East Asia, all member states of the South-East Asia and Western Pacific regions of the World Health Organization (Table 2). Forms of gynaecological cancers identified in the search are detailed in Table 3. Our focus on QOL included research with women in the defined geographical regions ever diagnosed with gynaecological cancer, from any stage pre-, during or post-treatment, or during their participation in interventions. Keyword searches were performed in six electronic databases: Web of Science, Scopus, Global Health (CAB Direct), PsycINFO (Ovid), EBMR (Ovid), and Medline (Ovid), with a tailored search strategy used in each, due to the variance in search interfaces across the databases (Table 3).

**Table 2:** Countries in South, South East and East Asia included in initial search

Country*	2021 World Bank income classification	World Health Organization Member State Region	Number of articles and studies included in review
Bangladesh	LM (low middle income)	South East Asia (SEA)	
Bhutan	LM	SEA	
Brunei Darussalam	HIGH (high income)	Western Pacific (WP)	
Cambodia	LM	WP	
*China*	*UM (upper middle income)*	*WP*	*18 articles *[36, 42, 43, 45, 49, 50, 52, 59, 62, 64, 65, 67, 72, 73, 77–79, 83]
*Hong Kong*	*HIGH*	*WP*	*10 articles, 7 studies* [21, 33, 37, 40, 48, 53, 55, 56, 58, 76]
*India*	*LM*	*SEA*	*1 article* [74]
*Indonesia*	*UM*	*SEA*	*3 articles, 2 studies *[32, 41, 66]
*Japan*	*HIGH*	*WP*	*4 articles, 3 studies *[51, 54, 57, 63]
Laos	LM	WP	
Macao	HIGH	WP	
Malaysia	UM	WP	
Maldives	UM	SEA	
Mongolia	LM	WP	
Myanmar	LM	SEA	
Nepal	LM	SEA	
North Korea	LOW (low income)	SEA	
Philippines	LM	WP	
*Singapore*	*HIGH*	*WP*	*1 article *[68]
*South Korea*	*HIGH*	*WP*	*4 articles *[60, 61, 69, 80]
Sri Lanka	LM	SEA	
*Taiwan*	*HIGH*	*UNLISTED*	*8 articles, 7 studies *[34, 47, 70, 71, 75, 81, 82, 84]
*Thailand*	*UM*	*SEA*	*4 articles *[35, 38, 39, 44]
Timor-Leste	LM	SEA	
Vietnam	LM	WP	

^*^Countries from which studies were included in this review are given in italics

**Table 3:** Search strategy

	“reproductive cancer” OR “gyn*ecolog* cancer” or “women* cancer” OR “cervical cancer” or “fallopian tube cancer” OR “ovarian cancer” OR “vulvar cancer” OR “vaginal cancer” OR “endometrial cancer” OR “uterine cancer” OR “HPV” OR “human papillomavirus”
AND	“quality of life” OR “QOL” OR “women* experience*” OR “patient experience*” OR “survivor*”
AND	“Asia” OR “Southeast Asia” OR “South Asia” OR “East Asia” OR “Bangladesh” OR “Bhutan” OR “Democratic People’s Republic of Korea” OR “North Korea” OR “India” OR “Indonesia” OR “Maldives” OR “Myanmar” OR “Burma” OR “Nepal” OR “Sri Lanka” OR “Thailand” OR “Timor-Leste” OR “East Timor” OR “Taiwan” OR “China” OR “Philippines” OR “Republic of Korea” OR “South Korea” OR “Cambodia” OR “Lao People’s Democratic Republic” OR “Laos” OR “Viet Nam” OR “Vietnam” OR “Japan” OR “Malaysia” “Mongolia” OR “Singapore” OR “Brunei Darussalam” OR “Brunei” OR “Hong Kong” OR “Macao”
NOT	“cellular biology” OR “molecular biology” OR “neoplasm”

Inclusion criteria were peer-reviewed journal articles in English language, which focused on any aspect of QOL, including physical, emotional, social, and cognitive functions, as well as pain, discomfort, and other symptoms [9, 10], among women who had ever been diagnosed with any form of gynaecological cancer and who resided in any of the countries detailed in Table 2. Articles were excluded if they included or focused on: male participants; participants diagnosed with cancers other than gynaecological; Asian populations in non-Asian countries; participants with genital warts or other non-malignant genital diseases; the epidemiology of cancer (i.e. prevalence, incidence, distribution of infection and rates); one or more specific risk factors correlating with cancer incidence; efficacy of a specific drug or test; or health worker perceptions of QOL among women affected by gynaecological cancer. We also excluded studies conducted outside of the countries of focus or comparative studies of a number of countries of which only some were from Asia; those that were strictly biomedical or clinical; and those that only utilised clinical data or patient files (i.e., retrospective studies). We also excluded articles written in languages other than English, mostly Chinese. We estimated that we have excluded 3% of articles that may have addressed QOL among women in China, with any kind of cancer, for this reason.

### Article selection and data extraction

**Fig. 2:**
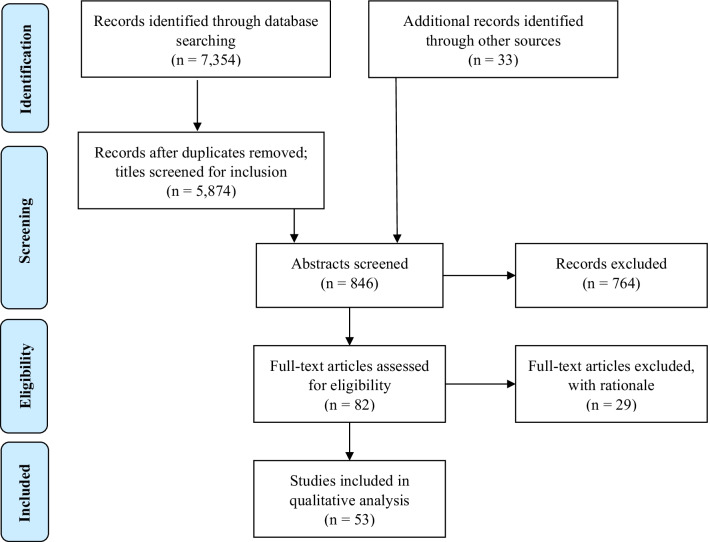
Literature search on quality of life among women affected by gynecological cancer in Asian countries (PRISMA flow diagram)

Following the removal of duplicate entries, CK identified and screened the titles of 5874 unique records (Fig. 2). After excluding articles that did not meet the inclusion criteria described above, CK then screened the remaining 813 abstracts, examined their reference lists, and identified repeat authors and publications. A further 33 articles were identified through this process. CK then prepared an annotated bibliography, mapping the key elements of the remaining 82 articles (Fig. 1). The key elements recorded in this bibliography included citation, abstract, key words, country of focus, type of cancer, methods, standardised tools used, and sample size. The key objectives and key findings of each article were also included.

The full texts of these 82 articles were then read and appraised by BRS, SSS and LRB to ensure that they met the inclusion criteria, to determine the breadth of content and key themes and findings related to QOL, and to develop a coding framework. We subsequently removed another 29 articles: we could not retrieve full text records for five; the other 24 were excluded, after reading the full text, because they did not meet the inclusion criteria. In our final analysis, we included 53 articles, and we identified four domains as most relevant in shaping QOL of women affected by gynaecological cancer: support; mental health; sexual function and sexuality; and physical health. A second level of synthesis was then undertaken by BRS using the four key domains and subthemes, and the relationships between key themes were explored by country, cancer type, participants’ position along the illness trajectory, and sociodemographic characteristics (Fig. 1). We present these results below.

## Results

### Description of included articles

Nine Asian countries were represented in 53 articles reporting on 47 studies (Table 2). Twenty-five articles discussed women’s QOL after diagnosis with cervical cancer; six focused on women with ovarian cancer and 22 articles included a range of gynaecological cancers, of which eight did not specify type or included ‘unknown’, ‘other’ or ‘mixed’ forms of gynaecological cancer. In 33 articles, the participants were post-treatment; 10 articles included women who were post-diagnosis; seven focused on women undergoing treatment; and three included women from different stages of their illness trajectory. Study sample sizes ranged from eight to 2268 participants. Nine articles reported on qualitative studies. Among the other studies, which were either quantitative (38 articles) and mixed methods (6 articles), 44 different standardised instruments were used to measure QOL (Table 1). The most common scales used in relation to cancer, mental health and sexuality, were the European Organization for Research and Treatment of Cancer (EORTC) Quality-of-Life Questionnaire (QLQ-C30), the Hospital Anxiety and Depression Scale (HADS) and the Female Sexual Function Index (FSFI). The thematic domains of these scales were incorporated in the four key domains of QOL that informed our analysis.

The QLQ-C30 is available in over 110 languages and includes 30 items within nine multi-item scales: five functional (cognitive; emotional; physical; role; and social); three symptom (fatigue; nausea and vomiting; and pain); and one global health and QOL scale [10]. Six single items assess appetite loss, breathing difficulties, constipation, diarrhoea, sleep difficulties, and perceived financial impact of disease and treatment. Ratings for each item, except global health and QOL, ranged from one (not at all) to four (very much). Global health and QOL were measured from one (very poor) to seven (excellent), and participants were asked: “How would you rate your overall health during the past week?” and “How would you rate your overall quality of life during the past week?”.

A three-step approach was applied to evaluate validity. The first involved correlations among various scales; the second, clinical parameters, treatment toxicity and patient ability to perform certain daily life activities; the third, responsiveness of the instrument to temporal changes in respondent health status [25]. The QLQ-C30 is the EORTC’s core instrument, incorporating domains relevant to different cancers and treatment modalities, and is often used with modules for specific cancers (e.g. 24-question cervical cancer module, called EORTC QLQ-CX24) [10]. The QLQ-C30 enables the comparison of QOL between cancer types; the disease-specific modules provide sensitivity to allow comparison between trials [10].

The HADS was designed in the United Kingdom to screen for clinically significant anxiety and depression in non-psychiatric patients [26]. It has 14 items within two subscales—anxiety and depression—but its validity and application in clinical research have been questioned [27–30], including to differentiate between anxiety and depression [27, 28]. Cosco and colleagues argued that the latent structure of the instrument is unclear and dependent on statistical methods used [27], while Mater and colleagues have questioned the translation, thresholds and cross-cultural application of the instrument [29].

The FSFI [31], initially validated in healthy women, has 19 items that measure domains of women’s sexual functioning, including arousal, orgasm, satisfaction and pain. Baser and colleagues tested the validity of the instrument for women affected by cancer [31], and found that its psychometric properties were strong enough to support its use to monitor sexual function and cancer‐related dysfunction among sexually active female cancer survivors [31]. We now turn to the primary themes identified in the publications reviewed.

### Support

Women affected by gynaecological cancer reported on the significance of social support in shaping QOL in 26 articles [21, 32–56], Support was provided by husbands and intimate partners, family members, friends, health workers, other women living with or who had survived cancer, and religious communities. Husbands and male partners were regarded as the central or preferred source of support in studies conducted in China, Hong Kong, Taiwan and Thailand [21, 34, 36–39, 55], and they provided women with emotional support, empathy and effective communication [21, 34, 36, 37, 55]. Although it is possible that some women were in same-sex relationships, articles only referred to husbands and male intimate partners. Indonesian cervical cancer survivors who participated in a psychosexual intervention (n = 16) reported that support and care from husbands was critical for intimacy and achieving female orgasm [41]. Partner support helped Taiwanese women (n = 11) adjust to changes to sexuality and ensured financial support if they had stopped working [34]. One study from China (n = 14) and another in Thailand (n = 208) reported that women’s partners sourced and paid for their medications [36, 39]. In two Thai studies intimate partners also undertook more housework and childminding than they had prior to women’s diagnoses [38, 39]. Male partners took women to medical appointments, encouraged them to rest, and attended to them when they were unwell [39].

Economic support for women undergoing cancer treatment, and their families, was emphasised in studies in Hong Kong, Thailand and China [21, 39, 42–45]. Hong Kong participants defined QOL as ‘having material resources or money’ [21]. Muslim women in Southern Thailand reported a high need for economic support during treatment, although the financial support they received fell short of their needs [44]. Compared with Thai Buddhists, these women had lower average incomes and were more likely to be engaged in unpaid work [44]. Participants in Northern Thailand reported that their families needed to work harder and borrow money to meet treatment costs [39]. In two Chinese studies, having low financial status was significantly associated with worse QOL [43, 45].

Women’s intimate partners were not always a source of support, and at times cancer resulted in the breakdown of relationships [21, 39]. In one Thai study, some partners were reportedly negative and unsupportive [39]. In Taiwan, additional financial reliance on an intimate partner created a level of dependence that made some women feel vulnerable [34]. Some married Indonesian women reported difficulties in negotiating cryotherapy for cervical cancer, because their husband’s permission was required for the procedure, likely because they were advised to abstain from sexual relations for 40 days following this treatment [46].

Women’s family members, friends and colleagues—usually other women—were important sources of support [36, 37, 39, 42, 44, 47–49, 55]. Chan and colleagues, for example, describe how family members and friends provided women with support by cooking nutritious meals and providing expensive foods, doing housework, childminding and accompanying women to medical appointments [55]. This was not always welcomed; in one study in China, women regarded the special meals cooked by family and friends as undesirable because they did not want to be treated differently [36]. In Taiwan women reported support from their children, who they perceived to be more thoughtful and affectionate [47]. In a comparative study of Thai Buddhist and Muslim women, regular meetings with family members were associated with a higher incidence of women’s psychological needs being met [44]. Similarly, family support mitigated mental distress and improved QOL for women undergoing treatment for recurrent ovarian cancer in China (n = 123) [49]. In Hong Kong (n = 8), visits during brachytherapy (focal internal radiation) treatment from family and friends helped them cope with isolation and immobility [48].

Studies in Hong Kong, China, Taiwan and Japan reported on support from health care providers, other women affected by cancer, and patient groups [36, 47, 48, 51, 55]. Health workers typically provided advice and information [55], but some also provided emotional support through reassurance and demonstrated empathy [47, 51]. Japanese women reporting on post-treatment care indicated that some doctors offered emotional support, but others were disengaged, seemed rushed and were non-communicative [51].

Emotional support offered by women’s peers was described in three studies [36, 47, 48]. In Hong Kong, other patients helped remediate the isolation and discomfort associated with brachytherapy [48]. In China, women who had not disclosed their diagnosis to others in their social circle particularly valued peer support [36], but as indicated in research in Taiwan, the death of peers was also a source of emotional distress [47]. Further, as illustrated in Taiwan, China and Thailand, social support was not always available, or was limited due to women’s non-disclosure or limited disclosure, due to fear of being judged by others [36, 39, 47]. Women reported feeling lonely and lacking someone to talk to (in Taiwan [47]), and social support was not always constant (in China [42, 52]).

Studies in Hong Kong, Taiwan and Thailand identified involvement with religious communities as providing spiritual support [21, 40, 44, 47, 53, 55]. In Hong Kong [21, 55], women’s religion was specified (mainly Christian and Buddhist). More than half of the Chinese women in one study had a formal religious practice, and faith provided them with a sense of belonging and purpose in life [55]. Access to private spaces for daily prayer times was reportedly an important consideration among Thai Muslim women [44].

Studies in China, Thailand, Japan, Indonesia and Hong Kong emphasised the provision of information to support women [32, 33, 35, 36, 41, 44, 50, 51, 53, 54, 56]. Psychoeducational and sexual health promotion interventions helped address women’s desire to expand their knowledge to cope with adverse impacts of cancer and treatment [33, 41]. Information that women in Hong Kong received, as part of research on a psychosocial intervention, helped them feel more supported and relaxed [33]. In Japan, prolonged health care seeking among women who lacked understanding of post-treatment side effects compromised their health [54].

### Mental health

The relationship between gynaecological cancer and mental health was discussed in 30 articles (29 studies) in the country contexts of Thailand, China, Hong Kong, South Korea, Indonesia, Taiwan and Japan [21, 34–37, 39, 44, 47–49, 51, 53, 54, 57–73]. Women suffered from anxiety and/or depression [47, 49, 51, 54, 57, 59, 60, 62–64], with a correlation between prior mental health conditions and distress associated with cancer. For example, Japanese women with low self-esteem, undergoing treatment for cervical cancer, were more likely to suffer from psychological distress than those with high self-esteem [57]. Chinese women with recurrent ovarian cancer had high rates of anxiety and depression prior to chemotherapy [49], and fear of cancer recurrence impacted women’s mental health [35, 37, 47, 51, 65, 66, 73]. In Indonesia, older women with gynaecological cancer of any type were more likely than younger women to fear recurrence [66]. Lower income and family history of cancer were also associated with fear of recurrence and treatment failure [66]. Manifestations of compromised mental health, reported across different studies and countries, included anger, tension and irritability [21, 36, 54]; depression [21, 47, 54, 59]; anxiety [44, 47, 48, 59]; frustration [36, 51]; feeling helpless [48]; rumination [47, 72]; negative thoughts [57]; lower self-esteem [37, 57]; suicidal thoughts [47]; social withdrawal [21]; and loss of appetite [47].

Six generalised risk factors compromised women’s mental health: age, in some research being younger [60, 61, 63, 67], in other studies older [21, 63, 66]; occupation [49]; education level [49, 62]; access to health insurance [49, 73]; and financial situation [34, 44, 49, 59, 60, 62, 66]. Risk factors for poor mental health directly related to cancer included: advanced stage of disease at diagnosis [59, 61, 62, 64]; treatment side effects—including pain, poor sleep quality, disability, infertility and physical changes [36, 37, 47, 48, 51, 54, 59, 73]; negative health care experiences [51, 54]; sexuality and body image issues [37, 60, 68]; recurrence [49]; and treatment modalities. In one Hong Kong study, women undergoing brachytherapy reported feelings of anxiety and helplessness [48]. In another Hong Kong study, compared with women who had had chemotherapy and radiotherapy, women who had undergone surgery experienced a deterioration in psychological adjustment [53]. Compared with a control group, Korean women with cervical cancer experienced greater anxiety about sexual performance [69].

Factors protective of women’s mental health and QOL included younger age at diagnosis [21, 70]; being married [58]; higher level of education [49]; increased capacity to work [53]; higher self-esteem [57, 70]; self-efficacy [64]; early stage diagnosis [21, 64, 65, 67, 71]; greater time elapsed since treatment [53, 58, 65, 67]; better mobility to engage in daily activities [21]; and high degree of social support [39, 49, 66, 70]. In addition, religious beliefs about karma helped Thai women with gynaecological cancer cope with fear of death [39].

Several studies included a qualitative component that elaborated on how women coped with the psychological burden and changes experienced while living with gynaecological cancer. Taiwanese women reported changing priorities and developing new interests post treatment [47]. Chinese participants noted that after diagnosis, while trying to keep optimistic and focused on living in the moment, they experienced a shift in priorities [36]. Women shared their experiences with peer patients, and this boosted their morale and provided encouragement [36]. In Hong Kong women reported gaining strength by getting through their treatment [21, 48]; Molassiotis and colleagues’ participants reported greater appreciation of their lives and their families, becoming more sensitive to the needs of others and gaining a more tolerant and positive outlook [21].

### Sexual function and sexuality

In 31 articles, women reported that having gynaecological cancer, and its treatment, altered sexual function and dampened sexual desire and response [21, 32–39, 41–43, 45, 47, 50, 51, 56, 58, 65, 68, 69, 71, 73–81]. This included loss of sexual pleasure, satisfaction and desire, including ability to achieve orgasm [21, 32, 34, 37, 38, 42, 43, 47, 73–77]; vulval and vaginal changes, such as lack of lubrication and elasticity, and atrophy [32, 34, 35, 47, 51, 73, 74, 77]; and difficult or painful intercourse [21, 32, 34, 35, 37, 39, 47, 69, 74, 77]. In two Chinese studies of cervical cancer survivors, roughly three-quarters of women reported sexual dysfunction [73, 78]. In another study in China, younger and higher educated women tended to be more concerned about their sexuality, compared with older and less educated women [45]. In a study of Indian cervical cancer survivors (n = 48), all women suffered from a decline in or loss of libido [74].

Certain treatment regimens had a pronounced impact on sexual function [69, 73, 77, 79–81]. For example, Taiwanese women who had chemotherapy reported worse sexual function than those who had not [75]. Chinese women who had radiotherapy were more likely to experience vaginal dryness and painful sex than those who had not [77] and, in another study, reported less frequent sexual activity than women treated with chemotherapy [79].

A sexual health intervention in Indonesia with women who had completed treatment for cervical cancer, and their partners, highlighted the potential for women’s sexual experiences to be improved through education [32, 41]. Participants received lubricant and attended information sessions covering: the aetiology of cervical cancer; treatment and side effects; strategies for resolving sexual problems; and exercises for relaxation and sexual fitness [32]. Women reported more mutual care, less painful sex, were better able to reach climax, and had more intimacy and greater confidence in their marital relationships [41]. In Hong Kong, women with gynaecological cancer participated in a psychoeducational intervention which included the theme ‘impact of treatment on body image and sexuality’, and consequently reported increased sexual activity, greater sexual interest from their partners, and increased intimacy [56].

The impact of gynaecological cancer and treatment on fertility was raised in one study in Hong Kong [21, 37]. This study emphasised that infertility could be perceived in either a favourable or negative light, depending on a women’s life stage and reproductive history. Infertility for some women contributed to low self-esteem and the decision not to marry; for women with children, infertility relieved them of the risk of having more [37].

Women across studies reported that compromised sexual function shaped their perceptions of sex and their sexual identities. In studies in Taiwan, Hong Kong, China and Thailand involving women with low to medium levels of education, participants avoided or had less frequent sex because they feared this would cause recurrence of the cancer [34, 35, 37, 65, 73, 77], or because they or their partners feared that cancer could be transmitted via sex [34, 35, 77]. Participants in China, Taiwan, Hong Kong and Thailand reported that their sex lives had ceased [36, 43, 47, 77], or that they were having less sex [21, 35, 38, 77]. Chinese participants experienced fewer sexual fantasies and masturbated less than ‘healthy women’ [76] but also, older women were likely to have worse sexual outcomes than younger women [76, 77]. One qualitative study of gynaecological cancer survivors in Taiwan described how women sought to maintain their sexuality and sexual function: taking vitamins or protein supplements; using vaginal lubricants; wearing makeup and wigs to feel more feminine; and engaging in sexual fantasies or using pornography to aid arousal [34].

Two studies in Hong Kong and China reported that women did not necessarily seek health care regarding their sexual function because they felt uncomfortable discussing their sex life with health care providers [37], feared judgement, or were embarrassed [78]. However, a study of inpatients in a Chinese hospital found that most were willing to discuss sexual issues and were interested in sexual health education [50]. More than half agreed that ‘doctors should raise the topic of sexual issues’; more than two-thirds were willing to raise the topic themselves if any sexual problem existed [50].

Numerous articles addressed gender identity and delved into women’s intimate relationships [21, 34–39, 47, 68, 69, 77]. Studies in Taiwan, China, South Korea and Singapore explored how women viewed their femininity and attractiveness [34, 47, 68, 69]. Women in Hong Kong and Taiwan regarded maintaining a sex life as important for maintaining family harmony and performing their normative gender roles [34, 37, 47]. Some women also reported privileging or being concerned about their husband’s sexual needs; for some this led them to engage in sexual acts that they did not find pleasurable [21, 34, 37, 47]. In Taiwan, some women engaged more often in non-penetrative forms of physical intimacy with their partners, such as kissing, hugging, holding hands, or non-physical forms, including endearments, mutual caring, and expressing gratitude and affection [34].

Two studies in Hong Kong and Thailand emphasised relationship changes due to gynaecological cancer. Some had separated from their husbands [37, 39]; and some husbands were having or were feared to be having extramarital relationships or sex with sex workers [21, 35, 38]. In one study in Taiwan, several participants reported that they had allowed their husbands to have a sex surrogate or had identified someone to take their place intimately and to care for their children if they died [34].

### Physical health

Twenty-one articles described the impact of gynaecological cancer and treatment on women’s physical health [21, 35–38, 42, 43, 45, 47, 48, 51, 53, 54, 68, 70, 75, 79, 81–84]. Of these, 16 related to continued effects post-treatment [21, 35, 37, 38, 45, 47, 48, 51, 53, 54, 68, 70, 75, 79, 81, 83]; and five involved women who were either post diagnosis [36] or were undergoing treatment at time of study [42, 43, 82, 84]. Whether women had surgery, chemotherapy or radiotherapy, or a mix of treatment, is determined by the diagnosis and staging of the cancer, although in general, as in a Chinese study, women reported that the more extensive the treatment of any kind, the greater the adverse side effects [36]. The three most commonly reported conditions were: pain (often in the back, shoulder, pelvic, uterus and sciatic regions) [21, 35–37, 45, 47, 48, 54, 68]; tiredness or fatigue [21, 36–38, 47, 48, 51, 54, 68, 75, 81–84]; and for women receiving radiotherapy and/or chemotherapy, gastrointestinal distress, including constipation, bloating, bowel obstruction, abdominal pain, diarrhoea and vomiting during treatment [36, 43, 45, 47, 48, 54, 68, 70, 75, 79, 81, 83, 84]. In a study in Taiwan, surgery was associated with higher rates of constipation [70, 75]. In another study [79], women treated with radiotherapy and then chemotherapy reported both lower QOL and more severe menopausal symptoms than women who had only chemotherapy. Physical impacts affecting women’s QOL included sleep difficulties and insomnia [36, 45, 47, 48, 82, 84]; urinary discomfort, unspecified ‘urinary disorders’ and incontinence [36, 51, 54, 68, 79, 81]; numbness, including of the extremities [36, 47, 51, 54, 84]; lymphedema [47, 51, 54]; weakness or loss of strength [36, 37, 51]; hot flushes and other menopausal symptoms [45, 54, 68, 82]; and restricted mobility [47, 48].

Women’s daily lives, including work and leisure activities, were affected by cancer symptoms, treatment, and side effects [21, 37, 38, 42, 51]. In one qualitative study, Japanese women depicted treatment side effects as akin to having a secondary illness [51]. The same study found that if post-treatment side effects were not recognised and acted on by women early, they could be prolonged and could worsen [54].

Women were not passive in face of side-effects and the management of illness. Living with gynaecological cancer motivated some women in Hong Kong to become more physically active post-treatment [37]. Similarly, women in one Taiwanese study engaged in self-care activities, including yoga, walking, foot baths and leg lifts [47]. In China, Taiwan and Japan, some women turned to alternative therapies to deal with physical effects of disease and treatment, including using traditional or herbal medicines, vitamins and acupuncture [36, 47, 51]. In Taiwan, auricular acupressure was used to manage chemotherapy side effects in ovarian cancer patients, and this reduced disturbed sleep, fatigue and appetite loss compared with women in the control group [82].

## Discussion

Of the 25 Asian countries included in our initial search, only nine are represented in this review, indicating the limited literature published in English on QOL among women living in these countries and affected by gynaecological cancer. Of the nine countries represented, five are classified as high income, three as upper-middle income and one as lower-middle income; no low-income Asian countries were represented. This may reflect the higher research capacity of high-income countries in the region, but it is of concern in terms of representation and because the incidence and mortality rates of gynaecological cancers are significantly higher in the middle-income Asian countries included in the review [85]. There is also imbalance in regional coverage, as most studies were conducted in East Asia. Additionally, as the majority of the quantitative studies included have relied solely on standardized instruments developed in Western contexts to explore QOL, they lack insights into the cultural nuances that offer a deeper understanding of the lived realities of Asian women’s experiences of gynecological cancer. Overall, the scope of the published literature indicates the need for further investment in understanding QOL of women affected by gynaecological cancer, wider geographical coverage and a focus on middle-income and lower-income countries, which constitute the majority of Asian countries. Most studies (n = 25) focused on cervical cancer, the most prevalent female reproductive cancer in lower and middle income countries. This was followed by 22 studies on the experiences and QOL of women with various forms of gynaecological cancer (as described in their inclusion criteria). Lack of differentiation of type of gynaecological cancer limited our ability to expand on the differences between cancer types, treatments and prognoses, and how these might impact on women’s QOL. In addition, cancer survivors reported positive changes in life outlook and self-growth, and an appreciation of their relationships with others and the preciousness of life [36]. However, very few of the articles attended to the positive outcomes of cancer survivorship, highlighting the opportunity for further research in this area. Another limitation was the inability to compare outcomes between studies.

### Support

A key finding was that high levels of support typically improved women’s QOL, and lower levels of support were reflected in poorer QOL. The strong relationship between support and QOL was mirrored in other studies of QOL, for instance, among breast cancer survivors in Asian societies, and so was not specific to women affected by gynaecological cancers [86, 87]. What was particular is the degree to which women can and do receive support when they are reluctant to disclose their diagnosis to potential support networks, likely associated with the stigmatization of gynaecological cancers compared with other cancers [39, 88]. The articles also indicated that narrow support networks for women with gynaecological cancer left women more dependent on their male partners for support, and desiring more supportive care and attention from health professionals.

Asian women affected by gynaecological cancer relied on various people for support including: husbands and intimate partners; family and friends; health workers and peers; and religious communities. The nature of support was: financial; emotional and psychological; informational; physical; spiritual; and practical (including performing domestic duties, accompanying women to medical appointments, and assisting to obtain medications). Consistent with a review on QOL among women affected by breast cancer, we found social support, financial support and support in accessing information to be key components of women’s QOL [89]. While religious communities and practices were identified as significant, there was little detail on their actual forms of support or how they enhanced women’s QOL.

Gaps in information were identified as a support deficit impacting negatively on women’s QOL. This highlights the need for greater attention to the provision of comprehensive patient education by health care professionals engaging with women and their families. The need for financial support for women living with gynaecological cancer confirms findings from a recent multi-country study in eight Asian countries, in which women survivors of cancer (all causes) with low socio-economic status experienced lower QOL [90]. The high levels of unmet need for financial support are significant given that no research from low-income countries was included; in these countries women’s need for financial support while living with gynaecological cancer will likely be greater. However, gaps in financial support and their negative impact on QOL suggest that women affected by cancer and living in a middle- or high-income Asian country are not necessarily financially secure. Gaps in financial support can be addressed via social welfare programs, and through improved health care coverage in health insurance schemes. These should be available to women throughout their cancer journeys, not merely at time of treatment.

### Mental health

Strong links exist between mental health and QOL, and between mental health and the degree of support women received. Manifestations of poor mental health included: depression and anxiety; irritability; frustration; negative rumination; suicidality; compromised self-esteem; and social withdrawal. Similar impacts in terms of psychological distress have been observed in other studies on mental health, including of women living with gynaecological cancer in Australia [91] and women living with other cancers in Asia [90]. Significant psychological impacts of gynaecological cancer were noted at diagnosis and prior to, during and after treatment, reflecting the different challenges and uncertainties women face at specific stages. The stage-specific concerns of women highlight the need for ongoing psychological care from diagnosis through to recovery and beyond.

Both risk and protective factors associated with psychological distress were reported. Risk factors independent of women’s cancer status and experiences were linked to either age at diagnosis or structural disadvantage. Risk factors for poor mental health directly associated with women’s experiences of gynaecological cancer included: being diagnosed at an advanced disease stage; negative health care experiences; disease symptoms and treatment side effects; impact on sexuality and body image; infertility; and recurrence.

Protective factors which supported women’s mental health and QOL included: younger age at diagnosis; diagnosis at an early stage of disease; higher levels of self-esteem and self-efficacy; increased capacity to work; high mobility; a high degree of social support; religious faith; and greater length of time since treatment. Specific coping mechanisms were also described. Some women reported a greater sense of appreciation of their lives and experienced a reorientation of life priorities. Relationships with peers with similar experiences proved important for maintaining mental health. The existing research provides a strong starting point for further research into how specific interventions could be developed to promote the mental health of women affected by gynaecological cancers in different Asian settings.

### Sexual function and sexuality

Gynaecological cancers have enormous impacts on sexual function, sexuality and intimate relationships and associated decreases in women’s QOL. Global reviews of sexual function among women affected by gynaecological cancer, and research conducted with women in non-Western contexts other than Asia, confirm that the impacts of cancer and its treatment on women’s sexuality are a crucial determinant of QOL [92, 93]. The impacts on Asian women’s sexuality were fundamentally shaped by the physical realities of the cancer and treatment effects, which directly affected women’s reproductive and sexual organs, and by cultural constructions of femininity, female sexuality and appropriate sex roles within heterosexual marriages. Physiologically, women widely reported: declines in sexual desire; loss of satisfaction; inability to achieve orgasm; shrinking or shortening of the vulva and vagina; vaginal dryness; and difficult or painful sex. Addressing changes in intimate relationships, sexual function and sense of sexuality was important to women affected by gynaecological cancer, and women drew on a range of strategies to address these concerns. These strategies were culturally embedded and ranged from choosing sexual surrogates for their husbands, adopting sexual practices other than intercourse, and using pornography and other sex aids to assist with arousal.

Only one study reported any interventions to address women’s sexual function and their desire to resume successful sexual relationships. Afiyanti and colleagues’ study [32, 41] in Indonesia demonstrated the potential for marked improvements in women’s sexual function and QOL. The success of their intervention counters popular notions that Asian cultures, in particular Muslim cultures, are sexually conservative, and illustrates how couples can effectively work together to address intimacy challenges. Clearly, there is a wide need for interventions of a similar nature aimed at providing culturally grounded responses that promote sexual health and function among women affected by gynaecological cancer.

### Physical health

Women with gynaecological cancer in Asia experienced extensive treatment side effects, and these side effects impacted negatively on their QOL and ability to function in their daily lives. The most pronounced side effects were pain, fatigue and gastrointestinal distress. Many women reported side effects that worsened as treatment extended. Specific side effects were associated with specific treatment regimens. For instance, in the studies, women were more likely to report gastrointestinal distress in association with chemotherapy than other modalities of care, and pain was more strongly associated with surgery. For some women, side effects associated with treatment were experienced as akin to having another disease, or as impacting more significantly on wellbeing and QOL than the physical symptoms directly caused by cancer. These findings point to the necessity of dedicated management of treatment side-effects, as well as the initial cancer disease, to protect women’s QOL.

Women reported a number of physical conditions affecting their QOL: sleep difficulties; urinary disorders and incontinence; numbness; loss of strength and reduced mobility; hot flushes and other menopausal symptoms. These findings are consistent with Kayl and Meyers’ review of the side-effects of chemotherapy in ovarian and breast cancer patients, in which they described the impact of side effects on women’s quality of life as “tremendous” [94]. Common forms of self-care women practiced to ameliorate the impact of physiological symptoms across various Asian societies included the use of acupuncture, herbal medicines and complementary medicines alongside biomedical care. This highlights the importance of medical pluralism in Asian women’s health seeking strategies.

## Conclusions

In the published English-language literature on QOL among women with gynaecological cancers in South, South East and East Asian countries, sexual function and sexuality featured most prominently (31 articles), then mental health (30 articles), support needs (26 articles) and physical health (21 articles). Our findings highlight the need for reforms in the delivery of health and patient care services for women, to address the interrelated domains of psychological health, sexual health and physical health. Such reforms should also attend to the differential support needs of patients across the disease trajectory.

Significant gaps exist in the geographical scope of existing research, emphasising the need to expand this research. We identified a dearth of research on QOL of women affected by gynaecological cancer in lower-middle and low income countries, even though women in such settings are both vulnerable to developing gynaecological cancer and have fewer resources to draw upon once diagnosed. The domains of support, psychological distress and mental health, sexual function and sexuality, and physical health should be included in the adaption of QOL scales. The range of gynaecological cancers included in studies of this type in Asian settings also requires extension, and where multiple cancers are included in single studies the reporting of research results should distinguish between the effects of different forms of gynaecological cancer on women’s QOL.

The inclusion of qualitative, mixed and quantative studies in this review highlighted the value of mixed methods to understand what constitutes QOL for woman affected by gynaecological cancer and the value of qualitative analysis to explain how the varied components of QOL are shaped by culture. Mixed methods and qualitative approaches are particularly salient in non-Western contexts, given that the standardized instruments typically utilized to measure QOL lack cultural grounding.

Further research on women’s sexual function and recovery of sexual health is necessary. Ideally such studies should be linked with appropriate interventions designed to improve women’s sexual function and QOL. The need for further research on mental health as a component of QOL is also evident. We suggest exploring cultural constructions of mental health and wellbeing in different Asian societies to inform interventions relevant to women’s worldviews and sense of personhood. How Asian women manage the side effects of treatment and physical symptoms of gynaecological cancer has also been neglected. Research has the potential to illuminate how and why Asian women use complementary and traditional medicines to promote their physical health and QOL.

Research on the long-term impacts of gynaecological cancer on women’s financial status and its resulting impact on QOL in Asian societies is also needed. Likewise, further research focusing on the relationships between social and economic support and psychological distress would enable a better understanding of how women’s QOL is determined by the interaction of social, structural and biomedical factors. Additional inquiry into Asian women’s experiences of cancer-related stigma, including the internalization of stigma, is crucial to address women’s social support needs and to tackle stigma. The significance of religion in promoting QOL and in providing support is another key area for further research, particularly in Asian societies where religious faith and practice remain central to daily life and worldviews. Research into QOL among women affected by gynaecological cancer in Asian societies is in a formative stage, and many gaps still need to be addressed. Filling these gaps has great potential to inform future practices and interventions to promote women’s QOL.

## Data Availability

Not applicable.

## Appendix 1: Summary of included articles

**Table:**

Authors	Date	Country	Cancer types	Participant status	Study type	N =	Standardised instruments utilised
Ratanasiri et al. [39]	2000	Thailand	CC	During treat; post-treat	Mixed	208 (208 in quant part & 79 in qual)	N/A
Molassiotis et al. [21]	2000	Hong Kong	OC, CC, UC (EC)	Post-treat	Mixed	62 (62 in quant part, 19 in qual)	WHOQOL-BREF; POMS; PAIS
Chan et al. [53]	2001	Hong Kong	OC, UC, CC, GTD	Post-treat	Quant	53	HAM-A
Molassiotis et al. [37]	2002	Hong Kong	OC, CC, UC (EC)	Post-treat	Qual	18	–
H Zhao et al. [83]	2003	China	OC, GTD, OTHER	Post-treat	Quant	216 (191 patients & 25 nurses)	QLQ-C30
CWH Chan et al. [55]	2001	Hong Kong	CC, OC, UC (EC & CORPUS)	Post-treat	Qual	18	–
Kritcharoen et al. [38]	2005	Thailand	CC	Post-treat	Mixed	194 (97 couples in quant part & 12 (6 couples) in qual)	N/A
Somjai & Chaipoom [44]	2006	Thailand	CC, OC & UNKNOWN	Post-diagnosis	Mixed	90	N/A
So & Chui [48]	2007	Hong Kong	CC	Post-treat	Qual	8	–
Park et al. [69]	2007	Korea	CC	Post-treat	Quant	860	QLQ-C30 QLQ-CX24 NHSLS
Tangjitgamol et al. [35]	2007	Thailand	CC	Post-treat	Mixed	105	N/A
Ding et al. [42]	2007	China	OC	During treat	Quant	61	FACT-O PSSS
Lai et al. [40]	2009	Hong Kong	CC	Post-diagnosis; Post-treat	Quant	173	WHOQOL-BREF
Kobayashi et al. [57]	2009	Japan	CC	Post-treat	Quant	60	HADS; FACT-G; SES
Hsu et al. [81]	2009	Taiwan	“UTERINE CERVICAL CANCER”	Post-treat	Quant	202	QLQ-C30
Kim et al. [60]	2010	Korea	CC	Post-treat	Quant	828	HADS; QLQ-C30; QLQ-CX24; MQOL
Tang et al. [76]	2010	Hong Kong	CC, UC, OC, MIXED	Post-treat	Quant	134	DSFI; MS; SSS; BRCS
Suzuki et al. [63]	2011	Japan	CC, EC, OC & OTHER	Post-diagnosis	Quant	214	HADS
Oshima et al. [51]	2011	Japan	CC, UC (EC), OC, VUC	Post-treat	Qual	28	–
Song et al. [80]	2012	Korea	CC	Post-treat	Quant	81	FSFI
Zeng et al. [77]	2012	China	CC, OC, UC, VUC	Post-treat	Quant	156	N/A
Xie et al. [65]	2013	China	CC	Post-diagnosis	Quant	194	MOS-SF-36
Ding et al. [52]	2013	China	CC	Post-diagnosis	Quant	106	FACT-CX; SOC-13
Li et al. [79]	2013	China	CC	Post-treat	Quant	2,268	QLQ-C30; QLQ-CX24; SAQ
Oshima et al. [54]	2013	Japan	CC, UC (EC), OC, VUC	Post-treat	Qual	28	–
ZM Zhao et al. [67]	2014	China	CC	Post-diagnosis; Post-treat	Quant	194	EQ-5D
Yang et al. [64]	2014	China	CC	Post-diagnosis	Quant	224	HADS; HHI; LOT-R; GSES
Chow et al. [33]	2014	Hong Kong	CC, UC, OC	Post-diagnosis	Mixed	26	SVQ; FACT-G MUIS HADS MOS-SSS
Ding et al. [36]	2015	China	CC	Post-diagnosis	Qual	14	–
Li et al. [70]	2015	Taiwan	CC	Post-treat	Quant	110	QLQ-C30; SES; MOS-SSS
Lee et al. [34]	2015	Taiwan	CC, OC, UC (EC)	Post-treat	Qual	11	–
Shao et al. [72]	2016	China	CC	Post-treat	Quant	95	PANAS; RRS; ERQ
W Zhou et al. [73]	2016	China	CC	Post-treat	Quant	140	FACT-CX; FACT-Sp; FSFI
Afiyanti et al. [32]	2016	Indonesia	CC	Post-treat	Quant	106 (53 couples)	FSFI
L Zhou et al. [78]	2017	China	CC	Post-treat	Quant	173	
Daga et al. [74]	2017	India	CC	Post-treat	Quant	48	FSFI
Li et al. [75]	2017	Taiwan	CC	Post-treat	Quant	290	QLQ-C30; QLQ-CX24
Tsai et al. [47]	2017	Taiwan	CC, UC (EC), OC	Post-treat	Qual	23	–
Wu et al. [71]	2017	Taiwan	GC (UC, CC, OC & “other”)	Post-diagnosis	Quant	167	QLQ-C30; SDS
Liu et al. [62]	2017	China	OC	During treat	Quant	198	HADS; PSS-10; HHI; RS
Wen et al. [49]	2017	China	OC	During treat	Quant	95	PSSS; HADS; QLQ-OV28; QLQ-C30;
Shao et al. [43]	2017	China	OC	During treat	Quant	95	QLQ-C30; QLQ-OV28
Hsu et al. [84]	2017	Taiwan	OC, CC, VAC, US, EC	During treat	Quant	89	N/A
Thapa et al. [45]	2018	China	CC	Post-treat	Quant	256	QLQ-C30; QLQ-CX24
Chow et al. [58]	2018	Hong Kong	CC, UC, OC, OTHERS (NOT SPECIFIED)	Post-treat	Quant	225	SFAGIS; MOS-SF-36; PAIS-SR
Teo et al. [68]	2018	Singapore	GC	Post-treat	Quant	104	FACT-G; BIS
Hu et al. [59]	2018	China	GC	Post-diagnosis	Quant	394	HADS
Wijayanti et al. [66]	2018	Indonesia	GC (NOT SPECIFIED)	Post-treat	Quant	153	FCRI; ISEL
Kim et al. [61]	2018	Korea	OC	During treat	Quant	150	DS14; MSAS-SF; FACT-G
Chen et al. [50]	2019	China	GC (types not specified)	Post-treat	Quant	1192	N/A
Tsao & Creedy [82]	2019	Taiwan	OC	During treat	Quant	60	MDASI
Afiyanti et al. [41]	2020	Indonesia	CC	Post-treat	Qual	32 (16 couples)	–
Chow et al. [56]	2020	Hong Kong	UC, OC, CC	Post-diagnosis	Quant	191	HADS; MUIS; SVQ;

Cancer types—*CC*  cervical cancer, *OC*  ovarian cancer, *UC*  uterine cancer, *GTD*  gestational trophoblastic disease, *EC*  endometrial cancer, *CORPUS*  cancer of the corpus, *VUC*  vulva cancer, *US*  uterine sarcoma, *VAC*  vaginal cancer; participant status—*During treat*  participants were undergoing primary, neoadjuvant or adjuvant therapy for gynecological cancer; Post-diagnosis  participants had been diagnosed with gynecological cancer, but had not commenced therapy; Post-treat  participants primary, neoadjuvant or adjuvant therapy for gynecological cancer had concluded; Study type—*Mixed*  mixed methods research; *Qual*  qualitative research; *Quant*= quantitative research

## Abbreviations

EORTC: European Organization for Research and Treatment of Cancer
EORTC QLQ-C30: European Organization for Research and Treatment of Cancer Quality of Life Questionnaire 30 Version 3
EORTC QLQ-CX24: European Organization for Research and Treatment of Cancer Quality of Life Cervical Cancer Module 24
FSFI: Female Sexual Function Index
HADS: Hospital Anxiety and Depression Scale
QOL: Quality of life
QLQ-C30: European Organization for Research and Treatment of Cancer Quality of Life Questionnaire 30 Version 3
